# Remarks, Gleanings, and Extracts

**Published:** 1874-11

**Authors:** T. Curtis Smith

**Affiliations:** Middleport, Ohio


### Remarks, Gleanings and Extracts.

BY T. CURTIS SMITH, M.D., MIDDLEPORT, OHIO.

*The Therapeutic Uses of Aconite.*—This was the topic of a paper recently read before the Medical Society of London, by Dr. Brunton. He showed that the only aconite that could be relied upon was the *aconitum napellus*, which had been selected by Baron Antonius Störck as the true drug, "eam a reliquis separare quæ vera est." Dr. Brunton said that aconite, as used in practice, had fallen into an undeserved oblivion, and he quoted from Dr. Garrod's Materia Medica: "It (aconite) is at the present time not very often employed, or only by a very limited number of practitioners (p. 163). After detailing the history of aconite and its preparations, he said that the best and least dangerous was the tincture of the British Pharmacopœia. He brought forward this paper as an expansion of one of Dr. Fothergill's corollaries from his paper, recently read to the Society, on the "Depressants of the Circulation." He (Dr. B.) showed briefly the action of aconite in poisonous and in medical doses, that it acted as a powerful depressant and sedative to the heart and circulation, and that when death occurs it is frequently as in hemorrhage. He quoted Dr. Fleming, who stated that patients who had taken too large a dose, but recovered, felt as if dying from excessive loss of blood. Upon this power of the drug was founded the therapeutic value. When administered in medicinal doses, the general result was similar to blood-letting, but in a somewhat different way. Blood-letting weakens the force of the heart by diminishing pressure on the vessels, while aconite diminishes pressure on the vessels by weakening the force of the heart. The action of aconite was—1. To lower the heart's action; 2. To lower the lung's action; 3. To lower the temperature of the body; 4. To produce free transpiration; 5. To produce sleep; 6. To starve a too vascular area. In short, aconite was our best substitute for general and local blood-letting; that it was our best agent just in those cases where formerly the lancet or leeches would have been used. He also showed that the power which had been assigned to this drug by Störck (in 1761), and which had since been denied, was quite correct, *i. e.*, its deobstruent power, and in support he quoted a few of many cases he had in practice, in which sole reliance was put on aconite for cure of enlarged glands, cervical, mammary, tonsillar, chronic hepatic enlargement, etc., and with complete satisfaction in result. He had notes of cases of acute disease where aconite was used, such as pulmonary congestion, catarrhal fever, pneumonia in its early stage, laryngitis, bronchial catarrh, acute nephritis, acute general eczema, and the like; he also detailed his observations on the temperature of the body during the action of aconite, and showed how rapidly and steadily it was lowered. Details were given of local inflammation, as orchitis and inflammation of the knee-joint, treated by aconite. Its use in the early stage of eruptive fevers was mentioned, and he found it cut short attacks of parotitis, and was decidedly most beneficial in acute ophthalmia. After detailing many illustrative cases, notes of which were taken during the last five years, and calling the Society's attention to the mode of administering aconite, viz: in small and often repeated doses, from one minim to a quarter of a minim every fifteen minutes for the first hour, or two hours, according as the circumstances of the case might demand, and one minim every hour after, the action of the drug thereby was kept up without producing poisonous symptoms. Of course, the aconite was to be omitted as soon as it had done its duty, and other suitable treatment adopted. He gave the following as his conclusions, after a very extensive use of the drug, in private practice, extending over a period of fully five years:—1. The best preparation is the British tincture; 2. It is best administered in oft-repeated small doses; it is nearly tasteless; 3. Its use can be continued for weeks, as it is not cumulative; 4. It is our best antiphlogistic drug; 5. It is diaphoretic and diuretic "nec æger nec sudore debilitatus est;" 6. If it does not remove the products of inflammation, when these are formed, by its control it prevents their formation, and so saves the tissue from further injury, and prevents tissue change; 7. It is most decidedly deobstruent; 8. It has the advantage that it does not leave that excessive weakness which follows ordinarily the diaphoresis produced by other drugs, such as antimony.—*Medical and Snrgical Reporter.*

*On Syphilitic Insanity.*—Mr. H. H. Newington says, in the *Journal of Mental Science*, January, 1874:

Syphilitic insanity is a form that requires considerable definition, as I think will appear from the following considerations. Dr. Wille, as I gather from Dr. Addison's German Retrospect in the January number of the *Journal of Mental Science*, 1873, has been able to assign a syphilitic origin in 2.5 per cent. or 1.40th of his cases, and states that even then the average is underrated, from the difficulty of obtaining proper histories. I have turned over the tables of causes of insanity on admissions in the 1872 reports of

47 asylums (20 England, 9 Scotland, 2 Ireland, 14 America, 2 Colonial), and find six cases only noted, 1 in England, 1 Scotland, 3 America, 1 Colonial, being and average on total admissions of .1 per cent., or 1-900th. What is the cause of the discrepancy? It can't be that Germans are more prone to syphilis in its worst forms than we are. It must lie in the manner and degree in which the disease is recognized. Giving full weight to a possible over-eagerness on the part of the Germans, and to the fact that the statistics which give such a high percentage are drawn from the whole population of asylums, whereas ours are drawn from the admissions only, we must own that our recognition is very limited; in fact, the causation is almost always allowed only in such cases as the one I have just described. Take our text books, for instance. Such a recent one as that of Dr. Maudsley only deals with tangible intercranial disease as a cause. Still more recent, that of Dr. Blandford hints at nutrition changes produced by syphilis having a hand in causing insanity; but he also writes this: "Syphilitic insanity is usually spoken of as syphilitic dementia." I have been able to find in various works the following relations between syphilis and insanity: 1. Acute mental disease may occur coincident with or even preceding and following the eruptive stage. This is a rare form, and seemingly requires great brain vulnerability for its production. 2. As a companion to the tertiary stage may occur a condition that is found with other forms of meningitis, to be followed often by dementia. Dementia also occurs sometimes without any appreciable intervening changes; but then it would be impossible to say that syphilis unaided had been the cause. 3. We meet with cases caused not so much by a specific brain destruction as by the sequelæ of a syphiloma. That condition might be well described as syphilomatous insanity. The name would, at all events, tie one down to a precise diagnosis, and at the same time afford pathological information to a reader of statistical tables of insanity. 4. We again find syphilis existing in a relation to melancholia; not a specific one, but, on the other hand, one that requires careful investigation before it is admitted in any given instance. Of course we at once reject mere syphilophobia without any manifestation of the disease. But we admit the power of corporeal diseases, such as irregularities in the alimentary canal, to determine at least an attack of melancholia; indeed, our first anxiety is to find out some such trouble, and we often find that, by setting that to rights, we ameliorate the mental condition. Therefore, it is reasonable to include syphilis as an agent in producing this form of disease. Dr. Wille gives considerable prominence to melancholia as a special symptom of syphilitic insanity.—*Medical & Surgical Reporter.*

*Diagnosis of Heart Disease.*—Dr. George W. Balfour, in the Edinburgh *Medical Journal*, says:

A truly cardiac patient, one suffering from actual disease of the heart, as a rule comes to you complaining, not of that organ, but of one or other of the secondary results of his lesion. He complains of breathlessness or of dropsy, either or both of which may result from that lesion if it be uncompensated, or if the compensation be ruptured. If the patient complain of shortness of breath, as is often the case, you will find that this cardiac breathlessness presents certain distinctive features wherein it differs from pulmonary breathlessness, the most striking of these being the perfect tranquility of the breathing while the patient is at rest, at the same time that any exertion at once produces so anxious a desire for more air as can be expressed by no fitter term than the air-hunger of the Germans. The amount of lesion is not to be measured by this breathlessness, but its seriousness, as dependent upon the degree in which the compensation is ruptured, may certainly be so. The patient may only puff considerably in going up a hill or ascending a stair, or his shortness of breath may be so great as speedily to compel him to call a halt on attempting either of these feats; or it may be extreme as to prove distressing on making such perfectly trifling exertions as merely sitting up or turning in bed. At the same time there is no true dyspnœa, or difficult breathing properly so called; there is no obstruction either to inspiration or expiration; there may even be no curtailment of the air-space in the lungs, from any cause whatever; the breathing while the patient is at rest is perfectly quiet and natural; yet such is the difficulty, from cardiac causes, of getting the blood aërated, that the slightest exertion produces such a gasping inquietude as is extremely characteristic. This is one form of cardiac asthma, as it is termed; now and then we have another, in which the breathlessness, though not dependent upon exertion, is yet equally independent of pulmonary lesion. In this case the patient wakes gasping and alarmed from his first sleep; he has palpatation, occasionally pain (angina), almost always irregular action of the heart, which is always feeble; now and then the patient is sick, and sometimes vomits a mouthful or two. This form of cardiac asthma is mostly senile in character, and associated with muscular degeneration rather than with valvular lesion. It frequently arises from some slight gastric derangement, which reflexly affects the enfeebled heart in an injurious manner; and it is often the beginning of the end to those affected, the first intimation that the "pitcher is broken at the fountain," and that death has already seized the very citadel of life. Such patients, however, never come to you, you are always sent for to see them; and I have only mentioned this affectation now to illustrate the fact that exertion is not always necessary to produce cardiac breathlesness, and that even in this case the panting is characteristic, while the absence of pulmonary lesion marks its cardiac origin. To produce so-called cardiac breathlessness, however, it is not necessary to have actual cardiac disease. Breathlessness depends upon imperfect aëration of the blood, and in the absence of pulmonary lesion may depend upon lesion of the heart or of the blood itself. Even though a patient, then, presents all the characteristic symptoms of cardiac asthma, we must not therefore set him down as certainly laboring under cardiac-disease; he may be only anæmic. But inasmuch as anæma and cardiac disease frequently co-exist, the assured presence of the former, evinced by the bloodless condition of the lips, gums, etc., does not include the latter. The presence of breathlessness having the characteristic symptoms described, makes us certain that we have to do with a hæmic or cardiac lesion; which it is, we must determine by further inquiries.—*Med. & Surg. Reporter.*

In Dr. L. Dusart's "Experimental Researches and Therapeutical Action of the Phosphate of Lime," the following case is related, which, if reliable, is certainly remarkable, as manifesting its powers in some forms of deficient bone structure:

Clemence M., aged three and a half years, extremely small for her age; skin is pale and waxy; the eyes heavy, dull, and anxious; pupils dilated. The head is enormous, compared with the rest of her body, and developed transversely, flattened from above downwards; the anterior fontanelle, largely open, is very soft; the head nearly bald. The belly is so large that it falls over the thighs, of which it covers all the superior parts, and which are separated; the deformed limbs are completely fixed and in a state of flexion, bending under the pressure like a sheet of lead; they cannot be touched without extreme pain being given. The radius, ulna, knees, feet, femurs and tibia are all deformed, but it is principally at the thorax that the deformity is most marked. The child can only utter a few words, but her intelligence is singularly acute. She is unable to make the slightest motion, except with the arms. She was losing weight daily, and appetite had entirely failed.

On the 21st of June, treatment was begun by giving her each day three desert-spoonfuls of syrup, which represents 1 gr. 50 of the lacto-phosphate of lime each day. This dose was not increased subsequently. The details of the recovery cannot be given, but in ninety-two days there was a gain in weight of 1640 grammes. The appetite was immediately aroused. From the second day the child was clamorous for food; the strength returned with astonishing rapidity. On the third day she could move her head, and the vertebral column took part in this movement. At the end of the eighth day she got up alone in her bed. In three weeks she could stand up with the back supported. The profound deformity of the osseous system was gradually repaired, so much so, that by the 18th of September, she could walk a few steps unsupported. At this time the patient had undergone the most wonderful transformation ; she is rosy, gay, and smiling. The head is now quite developed in the vertical direction, and seems to have been remodeled, The frontal line, from being curved, has become straight. The stomach has diminished to half its size; the legs are straight, and the foot can be placed on the ground. Complete recovery was finally accomplished.

While this case is very remarkable and interesting, it is not more so than many others recorded; and the book leaves a very strong impression as to the extreme value of Dr. Dusart's preparation of lime. The cases reported are all from prominent French physicians.—*American Medical Weekly.*

[He also relates some cases where long union after fracture was rapidly hastened even after long failure to unite before its use. It is no doubt worthy of trial in those cases, for while it is simple, the cases are of the most annoying character—Ed].

*The Communicability of Consumption.*—One of the best tests of this would be in the marital relation. At the Clinical Society of London, Dr. Webster stated that he had the history of the results of twenty-nine marriages between women with more or less marked signs of consumption, who married healthy men, and of fifty-one marriages of tainted men who married healthy women. While only one of the husbands of the twenty-nine diseased wives became consumptive, eighteen of the fifty-one healthy women married to diseased husbands died from consumption. The eighteen women were the wives of nine husbands, one of whom lost four wives, one three, four two, and three one each. Dr. H. Weber gave an abstract of the histories of these nine husbands and eighteen wives, and then discussed the following points: 1. The communicability of consumption from husband to wife he did not regard as established, but as rendered probable; he could scarcely consider the results of his experience as merely accidental, although the risk of communication was probably not quite so great as it would appear from his cases. 2. The means of communication between husband and wife seemed to exists only rarely in the inhalation of the breath, though he did not regard this as impossible, but more frequently in the seminal fluid, either by direct absorption of the latter, or indirectly through the fœtus. 3. The suggestion made to him, that possibily the infecting husbands were tainted with syphilis, was not supported by the examination of the facts, either those relating to the husbands or those relating to the wives, including the *postmortem* appearances. 4. The rapid course of the disease in the wives manifested more or less the character of galloping consumption, while the affections of the originally diseased husbands were in all cases chronic and quiescent, but well marked, and leading in all cases but one to a fatal termination, though long after the deaths of the wives.—*Med. & Surg. Reporter.*

*Influence of Anæsthetics upon the Sexual Impressions of Females.* A physician called as an expert before a United States tribunal made the following declaration : " A woman under the influence of anæsthesia is more liable to conception than when sexual intercourse has happened by force, and I concur in the opinion of Dr. Beck, expressed in his treatise on medical jurisprudence, that women may conceive during anæsthesia. The relaxation it produces facilitates conception."

This point seems to me established; but I desire to add an observation which I have made in my practice, and one that it deeply concerns physicians to know. It is well known to-day that occasionally under the influence oi ether or chloroform an excitation of the sexual organs is produced and a feeling is excited in the mind by this sensation which may make a woman believe that she has been subjected to violence.

The first case of this nature which I witnessed myself occurred during a delivery. The woman, placed under chloroform, experienced sexual sensations so vivid that she accused me of having violated her and called on her husband for protection. But he had been with her all the time as well as a dozen women who had never quitted her chamber. In a second case I was administering chloroform to a woman to have a tooth extracted, but the physiognomy of the patient showed an expression of venereal excitement so pronounced that I hastened to call in her parents. On awakening she seemed astonished to see herself surrounded by her family, and clearly exhibited what her impressions had been.

On another occasion a lady of a certain age entered my ofice in a state of high excitement, and related that she had gone to her surgeon to have a trivial operation performed, to relieve the pain of which she had taken chloroform, and the surgeon had abused her while under its influence. I was persuaded that she had deceived herself, and on examining all the circumstances clearly proved to her that she had been subject to a delusion.

The moral is that physicians should never administer ether or chloroform except in the presence of witnesses.—*Revue Medicale,* Aug. 17, 1874.—*Medical Examiner.*

*Incontinence of Urine in Children.*—Dr. Henry Kennedy, in the Dublin *Medical Press*, says:

Of the medical means employed, blisters to the sacrum must not be forgotten. There could be no doubt, the author said, this means had succeeded. Of two cases in which he had employed it, it failed in the first; but in the second it was more successful, and stopped the infirmity for four months. The regulation of the quantity of fluids taken, and the time, the author considers of much moment; and he particularly advised against the use of tea. There was one measure, too, he thought of the greatest consequence, and that was the teaching the patient, when such was possible, the habit of retaining the water as long as possible in the daytime. By this means the sensihility of the bladder was lessened, and good was effected. The author observed that this plan was opposed to the one of taking up the child at night, which, though it diminishes the unpleasant effects of the infirmity, had no tendency to cure the complaint, but, as he thought, the very contrary. To two medicines only did the author allude, hydrate of chloral being one, and belladonna the other. There was already some evidence that the former had been of service, but it was not sufficient yet to establish its value. The latter, as a whole, had proved the most valuable drug yet used, and had cured a good many cases. Of two cases in which the author gave it, it cured the first, a boy of three and a half years of age. In the second, a boy of eleven, it has bettered him a good deal; but circumstances had prevented as full a trial of the drug as was desirable. In speaking of belladonna, the author adverted to the remarkable fact that children bore it in very much larger doses than adults. By gradually increasing the dose he had given it in very large quantities. It had rarely dilated the pupils, and then only for a short period. In prescribing it this point was not to be forgotton. There could be little doubt that the internal organs, especially the kidneys, were so active in childhood that the poison was very rapidly eliminated from the system.—*Med. & Surg. Reporter.*

*Life Sustained by Nutritive Enemata for a Period of Twenty-two Days.*—At the last meeting of the Bristol North District Medical Society, Dr. J. B. Whitaker, of Fall River, Mass., reported the following case:

A strong, muscular man, 32 years of age, strictly temperate in all his habits, on the 3d day of May last, drank, by mistake, about three ounces of very strong, caustic potash lye. Antidotes were administered as soon as possible, but the injury was so severe that for thirty-nine days the patient could swallow only the most dilute liquids, and nutrition was aided by injections of beef-tea, gruel, etc. At the expiration of this time, a complete stricture of the œsophagus was formed, which prevented all attempts at deglutition for twenty-two days following. Efforts were made to overcome the stricture by the use of bougies, but not even the smallest could be passed. The patient was seen by Dr. Knight, of Boston, in consultation: and after a careful examination with the laryngoscope, and vain attempts to pass an instrument through the stricture, an unfavorable prognosis was given. Subsequent efforts, however, to dilate the stricture were successful, and a small bougie was passed into the stomach, after which a larger one, so that the patient could swallow fluids, and was improving satisfactorily. Subsequently, an attack of pleuro-pneumonia set in, from which he died seven days after the stricture had been overcome. The following is a summary of the case: Thirty-nine days supervened from the time of the accident to the formation of a complete stricture of the œsophagus. For *twenty-two days, the patient was wholly sustained by nutritive enemata;* and for seven days previous to his death he was able to swallow liquid nourishment with tolerable freedom.—*Boston Medical and Surgical Journal.*

*Cerebro Spinal Meningitis.*—Dr. Dowse, in the *Medical Times & Gazette*, gives the following hints as to treatment:

1. It has to be considered how to relieve the vessels of the cord, and to equalize the action of the vasomotor system of nerves. Nothing appears to be of greater service in effecting this than the ergot of rye and belladonna. The former he has prescribed in decided doses, such as half a drachm of the powder every four hours; and the latter he has applied to the spine in the form of a belladonna paste, made by mixing the extract with one-third its weight of glycerine.

2. To check the reflex vomiting, small pieces of ice must be swallowed, not sucked, as the full effect of its sedative influence upon the stomach is thus obtained.

3. To relieve constipation, Dr. Dowse prefers the administration of a pill of the watery extract of aloes, for the reason that it acts upon the mucus membrane of the rectum, and dilates the hemorrhoidal veins.

4. To relieve sleeplessness, both chloral and bromide of potassium have proved ineffectual, but what he found of most service was a suppository of eight grains of the extract of conium.

5. One essential practical point must not be forgotten, namely, to keep the paralyzed bladder constantly free from urine. It is not sufficient to draw off the water night and morning, which is the course usually adopted, but a self-retaining cathether must be kept continually in the viscus.

6. In reference to diet, it ought to be both nutritive and stimulant from the first.

7. There is a stage in the treatment of this disease where quinine in large does becomes of the most signal value—at that crsis when exhaustion appears imminent; the skin covered with sweat; temperature 102° to 105°; pulse small, weak, and over 120. But more especially is quinine invaluable when rigors supervene, when it never fails to have a good effect. It must, however, be given in ten or twenty grain doses; and, if the stomach cannot tolerate it, must be introduced into the system by the rectum.

8. The abstraction of blood in any manner is not advisable.—*Medical & Surgical Reporter.*

*Change of Life.*—As regards the relations of the change of life to insanity, Dr. H. Sutherland closes a valuable paper (*West Riding Asylum reports,* vol. iii.; *British and Foreign Medico-Chirurgical Review,* April, 1874,) with the following conclusions:

1. That insanity occurring at the change of life is not usually caused by that condition *per se,* but is most frequently due to some other moral or physical cause coincident with that period.

2. That the age most liable to attack is forty-five years and two months.

3. That the onset of the insanity generally occurs one year after the cessation of menstruation.

4. That the married state does not seem to predispose to that disease.

5. Nor does the number of the patient's children have any predisposing effect.

6. The forms of insanity most common are melancholia, and, more rarely, mania.

7. There is a certain group of symptoms sufficiently characteristic to enable us to diagnosticate a case of climacteric insanity, independently of any knowledge of the history of the case.

8. The prognosis is decidedly favorable, recoveries being over 40 per cent. of those attacked.

9. The duration of the attack is usually more than three months, and less than three years. Complete recovery is not to be expected until twelve months after the commencement of the attack.

10. With regard to treatment, mild sedatives and aperients, a careful watchfulness of suicidal tendencies, and the observance of a quiet and regular course of life, are chiefly indicated.—*Boston Medical & Surgical Journal.*

*Castor Oil Vomited Up Forty Minutes After Being Injected Into the Rectum*—By H. M. Hearn, M. D., Statesville, Tennessee.—On the 30th of July, 1874, I was called in great haste to Mrs. E.

H——, aged 47, who was complaining of great nausea and incessant retching—vomiting a glairy mucus, and also everything that was taken into the stomach; hence we were forced to resort to the use of injections, both hypodermical and rectal.

There being considerable constipation, it was evidently necessary to open the bowels as speedily as possibly. For this purpose we used the ordinary injection—castor oil forming a part, and which was vomited up within forty minutes afterwards. The attention of Dr. Fletcher (who was present in consultation) was immediately called to the appearance of the ejected matter in the night-glass, when he carefully separated the oil from the mucus, and bottled near a tablespoonful, which he took to his office and thoroughly examined. It was, beyond all doubt, the oil which had been injected, as she had taken no oil into the stomach.

Her bowels were freely purged shortly afterwards.

She has been a very healthy woman until within the last twelve months, during which time she has been suffering some irregularity in her menstrual periods.—*Nashville Med. & Surg. Journal.*

[This reminds me of a case in my practice in 1868. A young man with obstinately constipated bowels was attacked with the neuralgia of the same. A large enema was given him, per rectum, of soap-suds with salt dissolved in it. In thirty minutes he vomited a half pint of the injected fluid. The bowels moved well.—*T. C. S.*]

*Diagnosis of Stone.*—Dr. Henry H. Head, physician to the Adelade Hospital, reports a case, in the *Irish Hospital Gazette,* July 15, 1873, in which ausculation was employed as an aid to diagnosis of stone in the bladder. He says:

"I sounded his bladder, and was pretty sure I detected a stone, but did not think the evidence absolutely conclusive, when it occurred to me to try ausculatation, to see if it would assist my diagnosis. I accordingly applied one end of an India-rubber tube to the top of the catheter with which I was examining him, and the other to my ear, and at once heard, with great distinctness, the instrument strike the stone." He afterwards performed many experiments with substances of various sizes and degress of hardness, placed in a bladder distended with water, and found the sense of hearing to be more delicate than the sense of touch. "Even a small piece of chalk, not larger than a pea, could be most easily detected; the slightest touch of the catheter or sound being conveyed to the ear, when it could not be recognized by the hand." The stethoscope "consists of a small vulcanized India-rubber tube, about eighteen or twenty-four inches long, to one end of which an ivory ear piece is attached, similar to that used for ear-trumpets; and into the other end is inserted a metallic plug, with a tapering end protruding, which should be pressed tightly into the canal of the catheter; or, if a solid sound is used, the end of the tube, without the plug, may be fastened to it."—*Boston Journal*, December 26, 1873.—*Examiner*.

*Pposphoric Acid in Urine in Cases of Disease of the Encephalon*. Dr. E. Mendelt has made a series of experiments on the above (Archiv. für Psychiatrice) and has arrived at the following results: The quantity of phosphoric acid excreted by the kidneys under the influence of brain disease, and compared proportionaly to the other solid principles of urine, varies considerably from 2.49 to 3.93 per cent. The substance is excreted in greater quantity at night than during the day. In the chronic maladies of the encephalon there is a decrease in the absolute quantity of phosphoric acid excreted every day as well as of the relative quantity in connexion with the other solid principles of urine. In cases of maniacal excitement there is an increase in the absolute and relative quantity of the substance. Increase in the quantity is also observed during attacks of epilepsy and apolexy, and after the administration of chloral and bromide of potassium. The decrease of the substance in chronic cases of the brain disease must be attributed generally to diminution of muscular activity dependent on the protracted course of the disease. In other cases it may be ascribed to the general weakness and exhaustion of the nervous system the result of imperfect assimilation.—*London Lancet*, Sept. 26, '74—*Clinic*.

*The Function of the Uvula*.—Dr. Horace Dobell, in the *British Medical Journal*, says:

Looking, to-day, into the pharynx of a patient suffering from a severe nasal catarrh, I saw the watery secretions from the back of the nose pouring down in a continuous stream from the tip of the uvula on to the dorsum of the tongue. It was evident that they were collected to this point from all the surrounding parts, and that the uvula acted as a conduct to bring them to the front of the epiglottis, whence they might be safely carried down the throat by repeated acts of deglutition; whereas, had it not been for the uvula, they would be liable to drip behind the epiglottis, and thus cause constant discomfort by getting into the larynx. This very simple but important function of the uvula has not, so far as I am aware, been noticed before, notwithstanding all that has been written about this odd little organ.—*Med. & Surg. Reporter*.

*Removal of a Needle from the Heart—Recovery*.—G. W. Callender (*Med. Chirurg. Trans.*, vol. LVI) relates the following case:

The patient, a man, æt 31 years, after a struggle in a tavern, missed a needle which he had placed in the left side of his coat, who nine days later came under Mr. Callender's care, complaining of beating of the heart and constant pain extending from left nipple towards the axilla and down the inner side of the arm as far as the elbow. The pain slight, fullness and a sense of resistance in the fifth intercostal space were the only symptoms present, but were deemed sufficient to warrant an exploratory incision, and as soon as the skin and superficial fascia had been divided, the extremity of the needle was distinctly felt. It moved with each impulse of the heart, describing a double curve, and was thus evidently fixed in the heart itself near its apex. Removal was effected without difficulty, and the patient made a satisfactory recovery, though he was kept in bed for several weeks as a matter of precaution.—*American Jour. of Med. Sci.*, for October, ps. 471-2.

*Maternal Impressions.*—There was an extended and very interesting discussion of this subject at the meeting of the British Medical Association. Mr. Clapperton, who read the paper, cited several curious cases, and thought that the phenomenon was not confined to human beings. Dr. Coyder, who had formerly been an unbeliever, stated that he changed his views after a remarkable case in his own practice. He amputated the finger of a man in the presence of his daughter, then one month pregnant. She was much impressed, and her child had the corresponding finger wanting. The general opinion of those taking part in the discussion appeared to be that these cases were more than coincidences, but some gentlemen dwelt on the fact that a great number of froights are received by pregnant women without any bad results, even when they dreaded them. Dr. Fothergill stated that his mother had feared that he would be born minus an arm, which, however, had not been the case.—*Boston Med. & Surg. Journal.*

*Local Treatment of Pulmonary Cavities by Injection through the Chest Walls.*—Dr. Wm. Pepper, in the *American Journal of Medical Sciences* for October, 1874, clearly demonstrates the practicability of this kind of topical medication in those cases where the cavities are clearly proven to exist and to be within reach through the chest pareties. In three post-mortems following the use of such injections, no evidence remained to designate the points of injection, and no serious results followed immediately or remotely the plan adopted, which was by inserting a fine trochar and injecting the fluid with hypodermic syringe or an aspirator. Benefit seemed to result in five out of the six cases reported.

*Turpentine in Pyœmia.*—Dr. J. Sinclair Holden relates the case of a workman in whom amputation of the fingers was rendered necessary by an accident. Gangrene supervened, a secondary operation was performed above the wrist, and was, in its turn, shortly followed by rigors, profuse sweats, sleeplessness, low delirium, subsultus, and stupor, the wound becoming sloughy and offensive. The man rapidly sank, in spite of free stimulation.

As a *dernier resort*, half-drachm doses of turpentine were administered in egg emulsion every four hours. After the third dose they were discontinued, as the pulse and temperature had fallen, and consciousness returned. The patient partook liberally of brandy and beef-tea, but on the following day all the æsthenic symptoms re-appeared, and the patient relapsed into a comatose condition. The turpentine was again had recourse to, and with the same happy effect. This time the improvement was permanent, and the patient made an excellent recovery.—*Lancet—Nashville Medical & Surgical Journal.*

*Excision of a Portion of the Spleen—Recovery.*—Dr. H. C. Markham, of Winthrop, Iowa, reports (*Medical Record*, Sept. 15, 1874), the removal of almost the entire spleen of an Indian, who had been wounded in an altercation with a white desperado. When called to the wounded man, he found the spleen, to the extent of three-fourths of its volume, projecting from a wound in the abdomen. The organ was already partially sphacelated from constriction at the edges of the wound and the extreme heat of the season, about thirty-six hours having elapsed since the infliction of the wound. The projecting portion of the spleen was removed by a common bistoury to nearly a level with the abdominal walls. The hæmorrhage, which was excessive, was with difficulty controlled. After applying cold-water dressings and giving stimulanis, the patient was left to die, as Dr. Markham supposed. About a year afterward, the Indian presented himself at the doctor's office, and, drawing aside his blanket, exhibited the cicatrix of the wound. His only statement was "Indian heap no run."

*Case of Habitual Costiveness of Eight and a Half Months between Fecal Evacuations*—Dr. Thos. D. Strong, of New York relates in the *American Journal of Medical Sciences*, October, 1874, an interesting case of this kind. The subject had been habitually constipated from infancy, the difficulty progressively increasing with his age till months would pass between evacuations. When such did occur, the process lasted from " two to four days, at which time he is sick and becomes much exhausted." The approximate weight of such dejections is " forty pounds." The man is a laborer, doing light work on a farm. This, with one exception, is the longest continued case of constipation recorded, viz: Dr. L. Valentin's lasting (*Bull. Des. Sci. Med., t. x.*, p. 74) nine months.

*A Difficulty in Fœtal Ausculation.*—Dr. J. Braxton Hicks calls attention to a point with regard to the diagnosis of pregnancy and the life of the fœtus, by means of the existence of the fœtal heart-sounds—which he had not unfrequently observed in the course of his practice, but which he does not remember to have seen in print—and summed up his observations as follows: First, that the number of vibrations of the abdominal muscles in a state of half-suspension can be distinctly counted, watch in hand; second, that their number and sound is so like those of a very rapid fœtal heart that they may be mistaken for them.—*Philadelphia Medical Reporter—Medical Examiner.*

*Sulphide of Calcium in Boils.*—Few practitioners have been so fortunate as not to have been annoyed and often foiled in the treatment of a patient's suffering from the foruncular diathesis. Dr. Sydney Ringer, in the *London Lancet* for May, recommends the sulphide of calcium as the most effectual remedy. It may be given in doses of one grain or more to an adult, three to six times a day, in sweetened milk, or the tenth of a grain to a child under a year old. The effect is marked in the speedy arrest of suppuration and removal of induration. The treatment is described as being equally efficacious in mammary abscess, but not in indolent bubo.—*Jour. Mat. Med.*

*Lancing the Gums.*—Dr. James Finlayson concludes a paper in the *British Medical Journal* (Sept. 18, 1874) on the alleged dangers of dentition, and the practice of lancing the gums, with the statement that the chief danger of the wholesale use of the gum-lancet lies in its embodying in practice a theoretical view of the ailment, and so tending to close the mind against further inquiry into the diagnosis, etiology and treatment of infantile disorders.

*Bromide of Ammonium in Acute Articular Rheumatism.*—After a trial of more than a year, Dr. J. M. DaCosta is convinced of the value of the bromide of ammonium in the treatment of acute rheumatism. He thinks the drug relieves pain, acts generally upon the skin, keeps up the action of the kidneys and lessens the tendency to internal inflammation. He gives it in scruple doses every three hours.—*Medical Record,* Sept. 15, 1874.

*Aspiration of Urine.*—Prof. Connor, of Cincinnati, reports a case of injury to the urethra, in a boy, by falling on a bar of iron. It was impossible to pass a catheter. The aspirator was used by introducing the needle over the body of the bladder, drawing 21 to 24 ounces each day. On the ninth day, urine passed per urethra. Patient recovered.—*Clinic.*

*Catarrh Snuff.*—Dr. E. C. Mann, of New York, recommends the use of a snuff composed of equal parts of finely pulverized camphor and white powdered sugar as an adjuvant to the various injections and sprays employed in the treatment of nasal catarrh. —*New York Medical Journal.*

*Tracheotomy in an Infant.*—A case is reported in the *Journal de Thérapeutique* (1874, No. 15,) in which a tracheotomy was performed on account of croup, upon a child only fourteen months old, the operation resulting in immediate relief and speedy recovery.

A CONTEMPORARY states that a fee of $150,000 was lately paid to an American surgeon (Dr. Williard Parker, of New York,) for removing a wen by means of the galvano-cautery.

---

